# Supplementary material for: Patterns of Intron Gain and Loss in Fungi
Source: PLoS Biol. 2004 Nov 30;2(12):e422. doi: 10.1371/journal.pbio.0020422 (PMC532390; doi:10.1371/journal.pbio.0020422)
Supplement: Table S1 — Also available at http://genes.mit.edu/NielsenEtAl/. (4.3 MB ZIP). [file pbio.0020422.st001.zip › NielsenEtAl/html/85.html]

AN4515.1.NCU05686.1.MG01134.1.FG02720.1


```
 CLUSTAL W (1.82) Multiple Sequence Alignments - Introns Inserted


Sequence 1: NCU05686.1	477 aa
Sequence 2: MG01134.1	439 aa
Sequence 3: FG02720.1	477 aa
Sequence 4: AN4515.1	435 aa
Alignment Length: 495 aa
Number Identitical Residues: 144 aa
Alignment Score (without introns) 8352


MG01134.1 	MKS-TILSIGAA-ALLSAQSVLAADG--LKCSLDKHCPKEAPCCGL1YGDCGTGAFCLGG
NCU05686.1	MRSTTVLTLGAATALLGAGNVLADDI--PTCSLDKKCPESAPCCSQ1YGQCGVGAYCLGG
FG02720.1 	-----MLRSLVAAALLGASSVAA-----IKCTRDSHCPEDSPCCST1YGECGVGAYCLGG
AN4515.1  	---MLRLQTALFFAYLAARSLAADDFTPPSCSLDEHCPKEYPCCSV1YGQCGTGAYCLGG
          	      *      * *.* .: * . :. .*: *.:**:. ***.  **:**.**:****

MG01134.1 	CDPRMSFSLDSCVPEPTCQSKKFTFN--SKDRIQDVSEYLGDASKADWVVTGEPLFSNGN
NCU05686.1	CDPRMSFSLDSCVPAPVCQDKTYKMDKASMSKIVDISEYLGDPSKADWVSQGEPLSYNDN
FG02720.1 	CDPTMSFSLDSCVPAPVCQDRKMKMN--SLDRIVDIGKYLGDSDKADWVAQGEPVVFKDN
AN4515.1  	CDPLMSYSLDSCAPMPVCESKSYKWE--NLDSAASNNEYLGNATESDWVYSGKLKVEDGN
          	*** **:*****.* *.*:.:. . :  . .   . .:***:. ::***  *:    ..*

MG01134.1 	LLLTMPPRSVGTVLSSTHYMWYGNVKAKMKTSRGRGVVTAFILFSDVKDEIDYEWVGVDL
NCU05686.1	TLLTMPKNSVGTVLASTTYMWYGSVKARLKTSRGRGVVTAFILMSDVKDEIDYEFVGSEL
FG02720.1 	VLLTMPKDSVGTLLSSTVYMWYGNVKARFKTSRGAGVITAFILFSDVKDEIDYEFVGTEL
AN4515.1  	LVLTMPKESTGSLIANNHYIWYGKIGAKIKSSRGAGVVTAFILLSDTKDEIDYEWVGSDL
          	 :****  *.*::::.. *:***.: *::*:*** **:*****:**.*******:** :*

MG01134.1 	ETTQTNYYFQGIPKY~DQSGNITG-TSNTFENYHEYEINWTPDEITWLVDGKKGRTKKRS
NCU05686.1	TTAQTNYYFQGIPTY~ANSGNIS--VTDTYSNWHEYEIRWTPDQIQWLVDGQVGRTKLKS
FG02720.1 	GDAQTNYYFQGITNY1QNSENIS--LSDTFANYHDYEIRWTPDKIEWWVDGKMGRSLDKK
AN4515.1  	KEVQTNYYFQGILDY1DNGGKSKVDGGNTYADWHTYEIDWTPEKIDWLVDGEVVRTLTKE
          	  .*********  *  :. : . .  :*: ::* *** ***::* * ***:  *:  :.

MG01134.1 	ETWNATAQQWDFPQTPSRVQFSIWPGGADTNPKGTVDWAGGAINWVDHPDLKDPGYYYAM
NCU05686.1	ETWNATANQWDFPQTPSRVQLSIWPGGAASNAQGTIDWAGGAIQWDSE-DIKNYGYYFAT
FG02720.1 	DTWNATSKNFMFPQTPSRVQLSLWPGGKEGNAEGTVSWAGGPIDWDHP-DIQKSGYYYAA
AN4515.1  	STFNETADRYEYPQTPSRMQLSLWPAGQASNAQGTIEWAGGEIDWDSE-DIKNQGYYAAY
          	.*:* *:..: :******:*:*:**.*   *.:**:.**** *:*    *::. *** * 

MG01134.1 	VSEVEIKCWDGSNGVGTNKGKTYYYNSARGTNDTVVDSDKNGILSSLQATGLDMNKG---
NCU05686.1	FGEITVQCYDAKSPPGTNKGTSYYYNSYRATNDTVVDSDKPTVLASFQGTGTDMDAGKPS
FG02720.1 	FSDVEIECFNAKTAPGTNSGKSYWYNNEAGTNNTIVDGDKRHTIASLMATGEDMDKGKKK
AN4515.1  	YSDITVECYDPPSDAKVSGDVSYDFKDDKGLESSVEITDNNTVLASLGATGLNMDLG---
          	 .:: ::*::  .   .. . :* ::.  . :.::   *:   ::*: .** :*: *   

MG01134.1 	-------------GDK-----------------SKPDAS--TTGVDIP-----SVPGGSG
NCU05686.1	GTKGGSASATKAGGSKGT-----------SAPNSIPGAG--AAPNQVPGGGSGSVSGGSG
FG02720.1 	EEKKKEDKSKDDDKDKDDKDKDKEDDDEDDEPATIPGGGNGAPTNDHGNGDDSSSGSGSG
AN4515.1  	-------------SNKTD-----------------------------------SSGNSTG
          	              .*                                     *  ..:*

MG01134.1 	-VGPGQAGTNQGGGQ----AAQPPDNCFSDNFVQKCGTGGSSKNAGGRVETPSILS----
NCU05686.1	SSSSGSDGGSSAGGDSGSGASSGSSACGMNGFSQSCDDGSGSGSGGSSTKNNGVKTRDVQ
FG02720.1 	SSSDNAPKGNPDGTDDT--EPADTTNCDTSSFNQECGKSGSSKSK-SEDSGNGSNAGSRN
AN4515.1  	SISNSTVPQNQGGTGN---IAGSSTGNGGSSSSDSSGGAFSQGTDDSTDGENAAPSERVL
          	: . .    .  *  .    .  .     ..  :... . .. . ..     .  :    

MG01134.1 	GSSAFAVVVAFAGLVML
NCU05686.1	GASALAAVVGIVGLMVL
FG02720.1 	GASALAIIIAGGALFWL
AN4515.1  	KGSFFAVLVAVVVLIIM
          	 .* :* ::.   *. :
```
